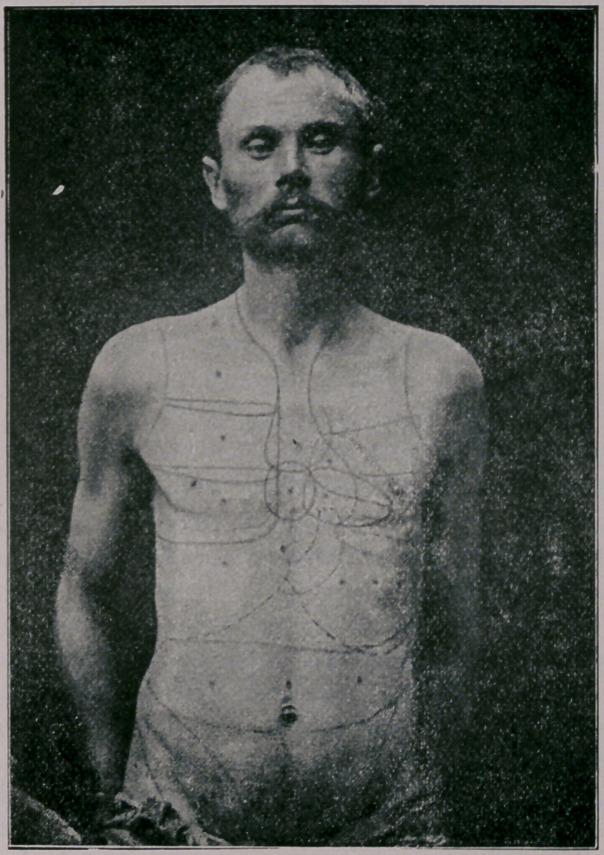# The Phonendoscope and Its Use

**Published:** 1897-08

**Authors:** O. L. Norsworthy

**Affiliations:** Houston, Texas


For the Texas Medical Journal.

### THE PHONENDOSCOPE AND ITS USE.

BY O. L. NORSWORTHY, M. D., HOUSTON, TEXAS.

[Read before the South Texas Medical Association in Galveston, Tex., May 14, 1897.]

BIANCHI, in association with Bazzi, a distinguished physicist of Rome, Italy, devised a much needed instrument, the phonendoscope, which, like all new discoveries and inventions, will plough its furrow into the fields of its greatest benefit. This invention, in my mind, will prove itself worthy of all favorable mention made of it for certain purposes.

The hope of the inventors was to obtain a simpler and more definite means of auscultating and percussing the various organs and pathological formations of the human anatomy. This, they realized, could be done only by transmitting the vibrations of sounds produced naturally or artificially in the human body, without diminishing the volume or altering the pitch or rhythm.

The phonendoscope consists of a cylindrical metallic capsule, which acts as a resonator, the lower end of which is closed by a thin ebonite disc. This disc is rendered slightly concave by a metallic spring, which is within the capsule.

The upper end is of metal, and has two metallic perforated knobs, also two openings into which are inserted two hollow metallic plugs, each bearing a soft rubber tube, tipped at the opposite end with hard rubber or ivory tips. The sides of the capsule are made concave for the purpose of winding the soft rubber tubes around it for convenience of carrying the instrument in a compact space.

There is a metallic rim which holds another thicker ebonite disc, in the center of which is a small button of the same materials, into which a small metal rod about two inches long, tipped with a bulb of ebonite, may be screwed. This rim is made so as to be used over and around the lower end of the capsule resonator or not.

The metallic staff, while not in use, may be screwed into the perforated metallic knobs on the top of the resonator.

The center of the ebonite plate in the lower end of the resonaator being rendered convex by the spring within, receives vibrations at that point especially, and comes in contact with the center of the outer plate, consequently receives the vibrations from the metallic rod when the latter is put into use.

The outer plate and small staff are only used when you desire to auscultate an area which is too small for the convex disc to come in close contact with, or for auscultatory percussion.

While auscultating, the instrument should be held by moderate pressure, either with or without the staff, to prevent too much tension of the springs. The two ear tubes must always be inserted into the resonator, otherwise it would not act as a resonator.

I have thus carefully and fully described the instrument, because only by accurately understanding its construction and mechanism can one realize its capabilities without actually testing it.

The instrument can be carried in an ordinary pocket, its weight being only about 8½ ounces, and it being very little larger than a large sized watch.

There being numerous makes of this instrument on the market, and some of them being substitutes of a very inferior kind, it would be advisable for the purchaser to be very careful as to what he is buying.

After comparing several different makes of this instrument, I am lead to believe that the "imported" Bazzi and Bianchi instrument is by far a greater improvement on the one made in the United States than the United States phonendoscope is on the ordinary Binanroe stethoscope.

On account of a law suit which is yet pending, the imported phonendoscope was patented in the United States in January, 1897, and is sold by a representative for the United States, a Mr. Feilchenfeld.

I think a thorough examination of the mechanism of the two instruments will explain to any one why the imported one transmits sound vibrations so much more acute and louder.

The phonendoscope made in the United States differs from the one I have herein described, which is the imported, by not having the spiral springs enclosed within the metallic resonator, and the ebonite discs being thicker.

The unnecessary thickness of the discs prevents the vibrations from being transmitted so freely, and the chain of connection is broken between the ear tubes and the receiver by the absence of the springs.

The uses of the instrument are manifold, serving as a most valuable aid to the clinical instructor and his students for accuracy in making diagnoses, as well as for economy of time; also to the general practitioner, for accuracy principally. By means of one or more pronged attachments, one or more sets of tubes may be attached, thus facilitating its use in the clinic rooms, when several persons may use it simultaneously.

Its use for facilitating physical examinations in timid women is worth more than its cost. Almost any cardiac examination can be made accurately over several layers of clothing, thereby relieving over-nervous and sensitive diseased women of the embarrassment of stripping off their clothing to the skin every time they get one of their hysterical heart spells. For the same reason the examining physician does not have to combat with the excited and unsteady heart action caused from the patient's embarrassed and excited condition.

Bianchi believes that the best results are obtained where only one bulb is inserted into the ear, but I have found the use of but one tube very unsatisfactory, and that for accuracy both tubes should be employed.

Bianchi divides the uses of the instrument into two great groups:

First,—The determination of sounds heard normally or pathologically inside the body by auscultation.

Second,—Sounds produced artificially by auscultation percussion.

Among the former he classifies the cardiac and respiratory sounds, gastric and intestinal peristalsis, the fœtal and uterine sounds, and crepitation of broken bones and joints.

He claims that the vibrations in the vascular walls produced by the blood current, and those due to contraction of voluntary muscles may be readily heard,—something which formerly could only be accomplished with the aid of the microphone. Under the latter group may be classed any sounds produced artificially over any part of the body.

For auscultatory percussion the outer plate and rod are attached, and the bulb of the rod placed over the organ to be examined. Vibrations are produced by gently striking the surface near the rod with the index finger.

Having ascertained the character of the tone produced by striking in the immediate neighborhood of the rod, we may proceed to strike away from it; or, as Banchi prefers, begin away from the staff, and strike centrifugally until you detect the change in the note, mark with pencil, and so outline the entire organ.

The force and manner of striking varies with different organs; some being deeper seated, and others less solid, are, of course, more difficult to percuss. Deep seated organs or tumors,—especially solid formations in the hollow viscera—necessitate vigorous striking, and vice versa. This, as in ordinary percussion, requires some practice to readily detect exact change in note.

Percussion of the chest walls should be parallel with the ribs if possible; otherwise, the sudden and successive changes from the freely vibratory bone tissue to the less vibratory soft muscular tissue may be confusing and uncertain.

In determining the sizes and situations of the various organs by this form of auscultatory percussion, the tip of the staff must be placed at definite points, which Bianchi has determined and mapped out. *Instead of giving a detailed description of these points myself, I shall refer you to his diagram.

---

*See New York Medical Record, Oct 31st.

It will be observed that several points are given for many viscera, which points must not include too much area, and the boundary lines obtained by the changes in the note must be determined at each point.

This method of examination is much simpler than it would seem to be from the description. It will be observed from the diagram that very few points are over bones,—the soft tissues being preferred,—as bone tissue vibrates so readily.

Comparative auscultation can be easily made by the use of two instruments upon corresponding areas, and a tube from either resonator. By compressing the tube from one instrument, and then the other, an exact comparative study of acoustic phenomena may be made. This is of especial importance in tracing the direction and extent of cardiac and aneurismal murmurs.

My somewhat limited experience with this instrument leads me to endorse its superiority over any other instrument used for auscultatory percussion, and for cardiac and fœtal examinations, though I am led to believe that the expectations raised by Bianchi are not as yet entirely realized. I find that it offers but few advantages over the ordinary Binaural Stethoscope for auscultation of the respiratory sounds. It has been found very valuable, however, in determining pericardial murmurs, and in fixing the upper level of fluid in hydrothorax and hydropneumothorax; also in determining deafness by some diagnosticians. None of such cases as these have I chanced to experiment with.

Bianchi's diagram shows that he has mapped out the lobes of the lungs and the ventricles and auricles of the heart,—neither of these have I done.

This instrument has been extensively used in Germany, and commended very highly by many good authorities. It has been used with great success by some of our Eastern diagnosticians, in locating and determining the sizes of some of the abdominal hollow viscera,—especially the stomach and colon.

Some of the diagnosticians consider its greatest field of usefulness in connection with the stomach and colon. I find that they may be very easily outlined by following out Bianchi's diagram, carefully observing by strokes, whether or not distended with gas or solid matter.

Case 1.—Mrs. J., white; 29 years of age; had been sick six months. She had been told by different physicians that she had heart disease, and received instructions from each to lead a very quiet and regular life.

This patient called at my office in December, 1896, telling me her own and the different doctors' diagnoses. After repeated examinations with the ordinary Gammann's Binaural Stethoscope, and finding a condition which I attributed to a reflex origin—probably from the cervix—I failed to give a positive opinion as to the heart disease. At that time, expecting a phonendoscope, which was then ordered, I succeeded in getting her to await its arrival.

After making numerous cardiac examinations of different healthy persons with the phonendoscope, I gave her several examinations, before being able to assure her absolutely that her heart was perfect in size, rhythm, pitch and intensity. Finally, to convince her, it was necessary that she compare her own heart with that of a lady friend of similar build. I dilated the cervix with Sea Tangle Tents, which gave her perfect relief. She has had no recurrence of nervousness, nor the cardiac palpitations since that time.

Case 2.—J. B., colored; 19 years of age; bellboy at hotel. Shows evidence of syphilis; has suffered from palpitation of the heart, and slight dyspnœa. Says he has suffered with it all his life. He had been told by different doctors that he had no heart trouble, which I confirmed, after making examinations with the stethoscope.

Several months afterward I made a phonendoscopic examination, and heard a peculiar soft, bellowing sound, following the first sound of the heart, just over the lower edge of the second left interspace, diffused very slightly toward the left shoulder, and in no other direction.

Since that time I have examined the boy from time to time, and always find the same murmur, which I diagnosed as being a slight stenosis of the pulmonary orifice, probably caused from congenital syphilis.

CASE 3.—Mr. A., Northerner by birth; has been living in the South about a year, and does office work. This young man looked somewhat pale and sallow. He was examined by the aid of the phonendoscope in January, 1897—that being the third time I had examined him, at intervals of from two to three weeks, for the same trouble. A pain over the liver, radiating downward to the right illiac region, and upward underneath the right scapula, covered his entire complaint.

At my first two examinations, which were made without the phonendoscope, I could find nothing abnormal in his condition, unless a possibly enlarged liver, of which I was not certain. The symptoms were relieved for a short time by the use of purgatives, after each examination.

On January 6 he was taken down with a hard chill, fever following. The third examination was made at this time, with the phonendoscope, which revealed to me distinctly an enlarged liver, and a very large gall bladder. All symptoms abated after treatment was directed to liver and gall bladder.

Case 4.—Mrs. L., a prostitute and beer drinker; has been treated for biliary calculi several years. I treated her about a year ago for the same trouble, at which time she passed several stones, which gave her longer relief than any previous treatment.

For practice—or an experiment, rather—I gave her a phonendoscopic examination in February last, at which time I could distinctly hear stone crepitation, upon manipulating the abdominal walls in the region of the gall bladder. They proved to be gall stones, by the passage of several small ones after a few days treatment.

Case 5.—Mrs. W., white; six months pregnant; had been under treatment for two weeks for fever. When I was called in March, 1897, no fœtal heart sounds could be heard, either with the ear alone or when aided by the phonendoscope, but a suppressed uterine souffle could be heard very indistinctly. Child was removed dead; placenta adherent and free to bleed.

Case 6.—Mrs. J., white, called me in April to tell her the cause of her large stomach, and stop her flow, which was considerable at the time, associated with uterine pains. She menstruated regularly every month, but the amount had been somewhat less since January, at which time she showed only a little pink water. After a very thorough examination I diagnosed pregnancy, but failed to detect any fœtal or uterine sounds by the ear or with the aid of the stethescope.

At my next visit I verified my diagnosis by hearing indistinctly both the fœtal heart sounds and the uterine souffle. After about two weeks' treatment, to prevent a then threatened abortion, I could hear neither fœtal nor uterine sounds with the phonendoscope. All symptoms indicated death of the fœtus, which was removed dead,—placenta being very badly diseased and disintegrated, having no attachments at all to the interior of the uterus.

Case 7.—Mrs. W., colored, called on me April 15 to remove a retained placenta, following a three and a half months' fœtus. I found the placenta projecting through the cervix, and the mother having no pains whatever about one hour after delivery of the fœtus. Failing to bring the placenta by expulsion, aided by traction on the projecting part with my right hand, I administered ergot,—to be repeated in one hour if needed, and to again call me if patient was not relieved of placenta in two hours.

When again called, I found her condition practically the same as when I left her, excepting that she was having very hard pains, with no good results.

Upon the application of the phonendoscope, I could hear a very indistinct rumbling noise, between pains, which I suppose was the uterine souffle. During the pains I could hear a peculiar, crackling, grinding sound, which I suppose was the crepitation, caused by muscular contraction, both abdominal and uterine. I decided that the placenta was adherent, chloroformed the woman, introduced my hand into the uterus, and found the placenta firmly attached to the right fornix by a space the size of the bottom of a glass.

In none of the first six cases here reported could I have been positive of my diagnoses without the aid of the phonendoscope.

In the first case, I was not absolutely positive of my opinion expressed against several other diagnoses, and had the phonendoscope not cleared up the conditions, the patient would not have allowed me to dilate the cervix.

I am sure that I would not have oeen able to tell you in this paper of having listened to a distinct pulmonic murmur, had it not been for the aid of the phonendoscope in case 2.

In case 3, I failed to detect the enlarged gall bladder at two previous examinations, without the phonendoscope,—which I feel convinced was the original trouble,— and that I could have detected it had I at that time possessed a phonendoscope.

In case 4, I had flattered myself with the idea that all gall stones had been passed after my last treatment, but the phonendoscope proved my flattery to be false.

In case 6, I am convinced that I could not have positively diagnosed pregnancy at the time without the aid of the phonendoscope, the case having such a contradictory history; and the death of the fœtus could not have been proven so conclusively; consequently, a longer delay for the removal of the fœtus would have been allowed, thereby jeopardizing the life of the patient by subjecting her to such dangerous conditions.

In conclusion, I will say that although my present experience with the phonendoscope will not enable me to corroborote and sustain all of Bianchi's claims,—and, admitting, as he himself does, that many of its uses may be accomplished with the binaural stethoscope,—nevertheless, an honest comparison of the two instruments will certainly convince any one of unbiased opinion that the phonendoscope is, in a great many respects, a distinct and decided improvement on the stethoscope.

## Figures and Tables

**Figure f1:**
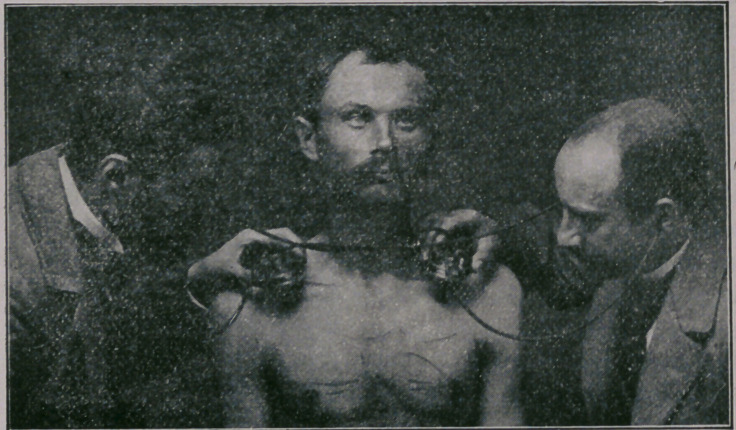


**Figure f2:**
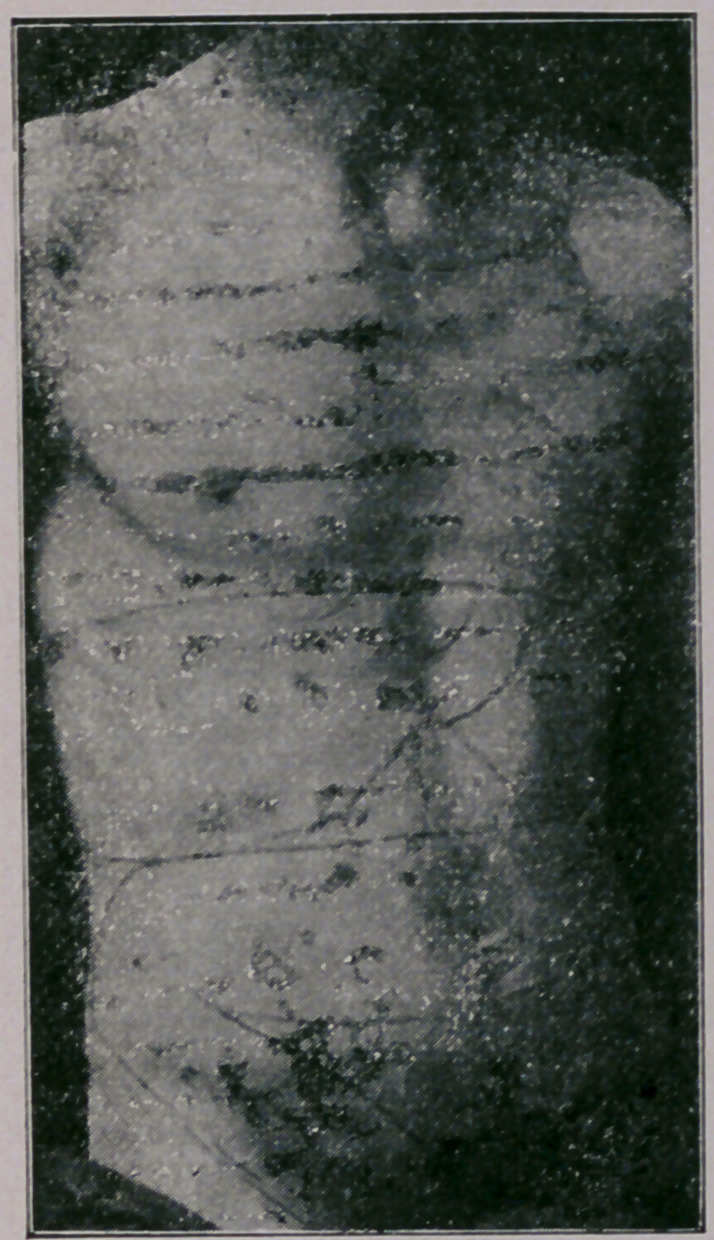


**Figure f3:**